# Early Results of Pilot Study Using Hepatitis C Virus (HCV) Positive Kidneys to Transplant HCV Infected Patients with End-Stage Renal Disease Allowing for Successful Interferon-Free Direct Acting Antiviral Therapy after Transplantation

**DOI:** 10.7759/cureus.890

**Published:** 2016-11-22

**Authors:** Juan F Gallegos-Orozco, Robin Kim, Heather F Thiesset, Jenny Hatch, Keisa Lynch, Thomas Chaly, Jr, Fuad Shihab, Faris Ahmed, Isaac Hall, Jeffrey Campsen

**Affiliations:** 1 Internal Medicine, Gastroenterology, University of Utah School of Medicine; 2 General Surgery, University of Utah School of Medicine; 3 Department of Surgery, University of Utah School of Medicine; 4 Division of Transplantation and Advanced Hepatobiliary Surgery, University of Utah School of Medicine; 5 Internal Medicine, Nephrology, University of Utah School of Medicine

**Keywords:** hepatitis c, transplant

## Abstract

Introduction: Hepatitis C virus (HCV) infection in kidney transplant (KTX) patients reduces long-term patient and graft survival. Direct-acting antivirals (DAA) are > 90% effective in achieving sustained viral response (SVR); however, DAAs are not routinely available to patients with end-stage renal disease (ESRD). The University of Utah Transplant Program developed a protocol to allow HCV-positive potential KTX recipients to accept HCV-positive donors' kidneys. Three months after successful KTX, they were eligible for DAA therapy.

Methods: HCV-positive patients approved for KTX by the University of Utah Transplant Selection Committee were eligible to be enrolled in this study. Patients consented for the use of HCV-positive donor organs. Three to six months after successful KTX, these patients were treated for HCV with interferon-free direct-acting antiviral regimens according to viral genotype and prior treatment experience.

Results: Between 2014-2015, 12 HCV-positive patients were listed for KTX. Eight patients were kidney only eligible, seven patients received HCV-positive deceased donor kidneys, and one received an HCV-negative organ. Currently, six patients have completed treatment, all have achieved sustained viral response (SVR), and one patient is currently awaiting treatment. All seven patients have functioning kidney grafts. Wait time for KTX was reduced amongst all blood groups from an average of 1,350 days to only 65 days.

Conclusions: HCV-positive patients with ESRD can successfully receive an HCV-positive donor's kidney. Once transplanted, these patients can receive DAA therapy and achieve SVR. Use of HCV-positive organs reduced time on the waitlist by greater than three years and expanded the donor organ pool.

## Introduction

Chronic infection with hepatitis C virus (HCV) is a leading cause of liver cirrhosis, liver failure, and hepatocellular carcinoma. It represents a significant public health burden with an estimated global prevalence of 3% (~170 million infected individuals) [1]. In the United States, the prevalence of HCV antibodies among the general population is ~1.5%; however, the prevalence is much higher among patients with end-stage renal disease on hemodialysis, ranging from 5.5% to 9.8% [1]. The liver-related morbidity and mortality of HCV among patients on hemodialysis are increased in comparison to that seen in the general population [2-3].

Kidney transplantation (KTX) extends survival and is cost-effective when compared with long-term dialysis [4]. However, KTX in HCV-positive patients is associated with increased risk of graft loss [5-7]. KTX recipients with active HCV infection have reduced long-term post-transplant survival and suffer from an increased risk of death from cardiovascular disease, secondary infections, and liver disease, as compared with KTX recipients who are not infected with HCV [5-6]. In spite of these differences, KTX, even in HCV-positive patients with end-stage renal disease (ESRD), improves outcomes compared to staying on dialysis.

As the demand for KTX far outweighs the supply of kidney donors, transplant centers have devised strategies to expand the donor pool. One such strategy is the use of kidneys from donors with HCV antibodies. Transplanting these organs into HCV-positive recipients has resulted in decreased waiting times.

We present here our experience with HCV-positive deceased kidney donors transplanted into HCV-positive recipients (with active HCV ribonucleic acid (RNA) replication), which resulted in a dramatic reduction in time on the list and has also allowed us to use the newer direct-acting antiviral agents to successfully treat the HCV infection after transplant.

## Materials and methods

After approval from the Institutional Review Board at the University of Utah (U of U) (approval #IRB_00081664), a single center prospective pilot study enrolled patients with a known history of HCV approved for KTX by the U of U Transplant Selection Committee into this study. Risks and benefits were clearly discussed with potential patients as well as the need for treatment with direct-acting antivirals after transplantation. Patients were then consented to receive HCV-positive donor organs.

Upon successful kidney transplantation and after immunosuppression stability, a time frame from three to six months, patients were treated for HCV with a combination antiviral therapy commensurate with their HCV genotype and prior experience with antiviral treatment pre-kidney transplant. Five patients infected with genotype 1 were treated with ledipasvir, 90 mg, and sofosbuvir, 400 mg (Harvoni®) (Gilead, Foster City, CA), with or without ribavirin (600 - 1,200 mg/daily), depending on patient tolerance, for a total of 12 weeks (the seventh patient also has genotype 1 and will receive the same medications). One patient with genotype 2b infection was treated with a combination of sofosbuvir, 400 mg (Sovaldi®) (Gilead, Foster City, CA), and daclatasvir, 60 mg (Daklinza®) (Bristol-Myers Squibb, Princeton, NJ) daily for 24 weeks.

## Results

A total of 12 patients with active HCV were listed for kidney transplant between the years 2014 - 2015 (Table 1). Only eight of the 12 listed patients were eligible to receive single organ renal transplants. The rest required multi-organ transplants and were, therefore, ineligible for the present study. As of April 2016, seven of those eight patients have received deceased donor kidney transplants. The eighth patient received a non-HCV infected organ but will still receive direct-acting antiviral (DAA) therapy per the protocol. This patient was listed with five years of dialysis time and, thus, was extremely competitive on the waitlist.

**Table 1:** Demographics, Relevant Kidney Transplant and Hepatitis C Parameters and Results of Antiviral Therapy. CNI: calcineurin inhibitor; DDA: direct-acting antiviral; HCV: hepatitis C virus; HTN: hypertension; KDPI: Kidney Donor Profile Index; KTX: kidney transplant; RNA: ribonucleic acid; SVR: sustained viral response; tCr: terminal creatinine.

	Age at KTX (years)	Gender	Recipient HCV genotype	Prior HCV Therapy	HCV RNA viral load at KTX	Donor KDPI	Donor age (years)	Donor tCr	Time on waitlist	Kidney disease	Recipient liver fibrosis score pre-KTX (stage 0-4)	Result of DAA therapy
Pt. #1	58	M	1a/1b	No	3,100,000	75%	48	0.92	56 days	CNI nephrotoxicity	Stage 2	SVR
Pt. #2	55	M	1a	Yes	2,100,000	51%	40	0.9	40 days	Lithium toxicity	Stage 0	SVR
Pt. #3	58	M	1a/ 1b	Yes	4,900,000	31%	24	1.08	9 days	Hepatorenal syndrome	Stage 0	SVR
Pt. #4	61	M	1a/ 1b	No	8,600,000	68%	40	1.3	109 days	Diabetic /HTN	Stage 2	SVR
Pt. #5	64	M	2b	No	23,000,000	91%	51	0.5	193 days	CNI nephrotoxicity	Stage 1	SVR
Pt. #6	56	M	1b	Yes	16,000,000	24%	25	1	42 days	Diabetic	Stage 3	SVR
Pt. #7	63	F	1b	No	Pending	73%	22	0.7	3 days	Lupus	Stage 0	Treatment pending

Six patients are male, ranging in age from 55 - 64 years. One patient is female age 63. Three patients had a history of failed interferon and ribavirin therapy while the other four are treatment naïve. Six out of seven patients have hepatitis C genotype 1 infection, while one patient has genotype 2 HCV. Liver histology prior to kidney transplantation showed no fibrosis in three recipients, Stage 1 in one recipient, Stage 2 in two recipients, and Stage 3 in the remaining recipient. No patient had liver cirrhosis (Stage 4 fibrosis).

Six of the seven patients completed DAA treatment and have all achieved a sustained viral response as defined by the absence of detectable HCV RNA (ribonucleic acid) 12 weeks after the end of treatment. One patient is pending treatment. The mean time between kidney transplant and initiation of antiviral therapy in these seven patients was 165 days (range: 109 - 209 days). Treatment within the first year after kidney transplantation decreases the risk of advanced liver fibrosis at the time of antiviral treatment.

Donor criteria resulted in a Kidney Donor Profile Index (KDPI) score ranging from 24% - 91%. Donor age ranged from 22 - 51 years. Donor organs were selected with a terminal creatinine with a range of 0.5 - 1.3 mg/dL. No other significant differences were observed amongst the donor criteria.

All of the patients treated on this protocol have functioning grafts at six months to one-year post-renal transplant. A significant reduction in the wait time for a kidney transplant was observed across all blood groups with an average of 1,350 days in non-HCV positive donors to only 65 days for those that received HCV-positive kidneys. The range of wait time in this population was three days to 193 days.

Details of the specific antiviral therapy regimen, duration, and results of treatment are described in Table 1. Treatment was overall very well tolerated with severe adverse events in two of three patients on ribavirin (severe anemia requiring blood transfusions and ribavirin dose reduction), but no patient discontinued antiviral therapy. The most frequent adverse events included fatigue (n = 3), headache (n = 2), anemia requiring blood transfusion and erythropoietin injections (n = 2), and nausea (n = 1). Both patients who developed severe anemia (hemoglobin < 8 g/dl) were on ribavirin. 

## Discussion

The liver-related morbidity and mortality of HCV among patients on hemodialysis are increased in comparison to that seen in the general population [2-3]. In a meta-analysis of 14 observational studies including more than 145,000 individual patients on hemodialysis, Fabrizi, et al. found that HCV positivity conferred an increased risk of all-cause mortality (relative risk (RR) 1.35), liver-related death (RR 3.8), and cardiovascular mortality (RR 1.26) [8].

Kidney transplantation (KTX) extends survival and is cost-effective when compared with long-term dialysis [4]. However, KTX in HCV-positive patients is associated with chronic rejection, transplant glomerulopathy, HCV-glomerulonephritis, and post-transplant diabetes, leading to increased risk of graft loss [5-7]. KTX recipients with active HCV infection have reduced long-term post-transplant survival and suffer from an increased risk of death from cardiovascular disease, secondary infections, and liver disease, as compared with KTX recipients who are not infected with HCV [5-6].

The negative effects of HCV on post-KTX outcomes were recently demonstrated in a meta-analysis of 18 observational studies including 133,350 KTX recipients. Compared to HCV-negative recipients, HCV-positive KTX patients had a 76% increased risk of graft loss (RR 1.76) and an 85% increased rate of all-cause mortality (RR 1.85) [7]. In spite of these differences, KTX, even in HCV-positive patients with ESRD, improves outcomes compared to staying on dialysis.

With the above information in mind, there has been a strong emphasis on treating patients with HCV and ESRD prior to KTX; however, up until five years ago, treatment strategies were limited to interferon and ribavirin, with less than ideal response rates and the risk of severe adverse events among this population. The advent of well-tolerated, interferon-free, direct-acting antiviral therapies has resulted in significant increases in sustained viral response rates (~95% for most patients) and allowed us to treat more patients with chronic hepatitis C. However, even with these newer therapies, treatment options for patients with ESRD are still limited. In February 2016, the FDA approved the combination of elbasvir with grazoprevir to treat patients with chronic HCV genotypes 1 and 4. This is the first and only regimen currently approved for use in patients with ESRD on dialysis [9].

We describe our experience with HCV-positive deceased kidney donors (D+) transplanted into HCV-positive recipients (R+) (with active HCV RNA replication), which resulted in a significant reduction in wait time on the KTX list compared to our institutional average. The benefit to our patients was not only an earlier KTX but also the ability to treat their chronic hepatitis C successfully within the first six months after transplant without deleterious consequences to their graft. The DAAs were effective and very well tolerated without severe adverse events or drug-drug interactions with immunosuppressive regimens, except for two patients who developed severe anemia (hemoglobin < 8 g/dl and required ribavirin dose-adjustment and transfusion of packed red blood cells). All of the patients who have completed their treatment course have gone on to achieve sustained viral response, the seventh patient is awaiting treatment.

There has been a concern that HCV-positive recipients of HCV-positive kidneys have worse clinical outcomes compared to HCV-positive recipients of HCV-negative grafts [10-12]. More recently, however, Scalea, et al. retrospectively reviewed the KTX experience at the University of Maryland and demonstrated that HCV-positive KTX recipients of HCV-positive grafts (R+/D+) spent less time on the kidney transplant list resulting in improved death-censored graft survival when compared to HCV-positive recipients of HCV-negative grafts (R+/D-) [13]. Of 1,679 KTX recipients, 195 HCV-positive recipients (R+) received transplants from HCV-positive donors (D+), 66 HCV-positive patients received an HCV-negative graft (D-), and 1,418 HCV-negative patients received an HCV-negative graft. The death-censored graft survival in the R+/D+ group was better than the graft survival in the R+/D- group, despite R+/D+ patients having higher rates of hypertension and African Americans. Waitlist times for the R+/D+ population was 318 days, compared to 570 days for the R+/D-, and 613 days for the R-/D- groups, respectively. On multivariate analysis, waitlist times were independently predictive of graft failure [13].

There is a suboptimal utilization of HCV-positive kidney donors in the United States. Kucirka, et al. characterized the use of HCV-positive kidneys for HCV-positive recipients by analyzing KTX data from the United Network for Organ Sharing (UNOS). Of 93,825 deceased donors between 1995 and 2009, HCV-positive kidneys were 2.6-times more likely to be discarded (p < 0.001). However, of the 6,830 HCV-positive recipients, only 29% received HCV-positive kidneys. Patients over age 60 (RR 0.86), women (RR 0.73), and highly sensitized patients (RR 0.42) were less likely to receive HCV-positive kidneys, while African Americans (RR 1.56), diabetics (RR 1.29), and those at centers with long waiting times (RR 1.19) were more likely to receive them. HCV-positive recipients of HCV-positive kidneys waited 310 days less than the average waiting time at their center, and 395 days less than their counterparts at the same center who waited for HCV-negative kidneys, likely offsetting the slightly higher patient (hazard ratio (HR) 1.29) and graft loss (HR 1.18) associated with HCV-positive kidneys [14]. More recently, Reese, et al. identified 3,273 HCV-positive deceased kidney donors from 2005 to 2014 from the UNOS database. Of these 6,546 kidneys, only 37% (2,400) were actually transplanted, 91% to recipients with HCV infection. The rest of the kidneys were discarded, although most were of good quality. Per their estimation, these discarded kidneys could have benefited ~4,000 patients and provided more than 12,000 years of graft life by five years after transplantation [15]. These data underscore the possibility of significantly expanding the donor pool by using all or the majority of the kidneys from HCV-positive donors. With the highly effective DAA currently available, there is even discussion of the use of HCV-positive organs to be transplanted into HCV-negative recipients, although this certainly would bring along significant medical and ethical challenges, such as purposely infecting a KTX recipient with HCV and the added cost of treating them after transplant [15].

The published experience of DAA therapy for HCV in the post-kidney transplant setting is very limited, including only two retrospective studies. In the only US experience, Sawinski, et al. reported on 20 KTX recipients with HCV treated with sofosbuvir-based interferon-free DAA regimens for 12-24 weeks [16]. The majority of patients were infected with HCV genotype 1 (88%) and 50% had advanced liver fibrosis (Stage ≥ F3) on pre-treatment liver biopsy. The median time from transplant to HCV therapy was 888 days (interquartile range: 341 - 1,621 days). There were no early discontinuations, and all patients achieved SVR at 12 weeks after the end of treatment. There were no episodes of graft rejection while on treatment and less than half of the patients required dose adjustment of their calcineurin inhibitor. Only three patients were treated with ribavirin and two of them required dose reduction due to anemia, including one patient that warranted blood transfusion for symptomatic anemia. Of note, only nine of the 20 patients were transplanted with HCV-positive kidneys. Follow-up was fairly short, and hence, no significant conclusions could be drawn regarding long-term clinical benefits from viral eradication. In the French experience, Kamar, et al. [17] reported on 25 KTX recipients treated with sofosbuvir-based DAA regimens for 12-24 weeks (six required ribavirin). The median time from KTX to DAA therapy was 146 months (range: 1 - 329 months); 76% were infected with HCV genotype 1 and 44% had advanced fibrosis (Stage 3 or 4 of 4). All patients achieved SVR. Treatment was safe and well tolerated without significant drug-drug interactions; there were no episodes of acute rejection and renal function remained stable. Similar to the US experience, follow-up was too short to draw strong conclusions regarding the long-term benefit of treating HCV infection in the post-KTX population. The authors did not specifically mention how many (if any) of the 25 recipients were transplanted with an HCV-positive donor.

The main difference between our current report and the US and French experiences is the prospective nature of our pilot study compared to the retrospective nature of those reports. All of our patients were transplanted with HCV-positive grafts, which was not the case in the two published series. Another important difference was the time from transplant to treatment; our patients started DAA therapy very early in the post-KTX period (mean: 165 days), compared to a median of 888 days in the US experience and a median of 4,380 days in the French experience [16-17]. Although our follow-up is very short, we predict that early eradication of HCV infection can result in significant long-term benefits to patient and graft survival.

## Conclusions

In conclusion, patients with chronic hepatitis C and ESRD on hemodialysis who accept HCV-positive kidneys derive benefits on two fronts. First, they spend much less time on the transplant waitlist, which can potentially result in improved graft outcomes compared to HCV-positive recipients of HCV-negative grafts. Second, having normal kidney function allows them to get early treatment for the HCV infection soon after transplant with excellent response rates, which could derive in long-term clinical benefits. Based on this successful pilot study, this protocol has become a standard of care at our transplant center.
